# Short Dental Implants (≤7mm) *Versus* Longer Implants in Augmented Bone Area: A Meta-Analysis of Randomized Controlled Trials

**DOI:** 10.2174/1874210601812010354

**Published:** 2018-04-30

**Authors:** Priscila N. Uehara, Victor Haruo Matsubara, Fernando Igai, Newton Sesma, Marcio K. Mukai, Mauricio G. Araujo

**Affiliations:** 1 Department of Prosthodontics, School of Dentistry, University of Sao Paulo, Sao Paulo, Brazil; 2 Dental School, Oral Health Centre of Western Australia, The University of Western Australia, Perth, WA, Australia; 3 Department of Dentistry, State University of Maringa, Parana, Brazil

**Keywords:** Dental implants, Survival rate, Bone tissue, Meta-analysis, Systematic review, Clinical trial

## Abstract

**Aim:**

The aim of this systematic review was to compare the survival rate and the marginal bone loss between short implants (≤7 mm) placed in the atrophic area and longer implants placed in the augmented bone area of posterior regions of maxillaries.

**Methods:**

Electronic search using three databases was performed up to May 2017 to identify Randomized Controlled Trials (RCT) assessing short implants survival with a minimal follow-up of 12 months post-loading. For the meta-analysis, a Risk Difference (RD) with the 95% Confidence Interval (CI) was used to pool the results of implant failure rate for each treatment group. For the marginal bone changes, Mean Differences (MD) with 95% CI were calculated.

**Results:**

Seven randomized controlled trials met the inclusion criteria, being included in qualitative and quantitative analyses. The RD between the short implant group and the control group was -0.02 (95% CI: -0.04 to 0.00), I^2^=0 and Chi^2^=3.14, indicating a favorable survival rate for short implant, but with no statistical significance (*p*=0.09).

**Discussion:**

For marginal bone loss, the mean difference was -0,13 (95%CI: -0.22 to -0.05), favoring the test group with statistical significance (*p*=0.002). The studies showed more heterogeneity for bone loss compared to survival rate. Short and longer implants showed similar survival rates after one year of loading, however the marginal bone loss around short implants was lower than in longer implants sites.

**Conclusion:**

Placement of implants ≤7 mm of length was found to be a predictable alternative for the rehabilitation of atrophic posterior regions, avoiding all the disadvantages intrinsic to bone augmentation procedures.

## 1 INTRODUCTION

Quality of life in adults can be highly affected by tooth loss as a consequence of compromised oral function, loss of social status and diminished self-esteem [1]. The oral rehabilitation using implants has a positive implication in the reestablishment of all these factors that affect the life of patients. Oral implant placement also provides a more comfortable and aesthetical treatment option for partial and complete denture wearers, being widely accepted by patients as an efficacious method for replacing missing teeth [2, 3].

Multiple tooth extractions induce a considerable reduction in bone height, mainly in the posterior jaws [4]. In the maxilla, the absence of teeth promotes sinus pneumatization and consequently vertical bone loss [5], whereas the presence of the inferior alveolar nerve in atrophic mandibles limits the length of implants [6]. Therefore, the rehabilitation of edentulous posterior regions using implants becomes complex when severe ridge atrophy is presented. 

Different surgical techniques enabling the reconstruction of maxillaries with reduced bone height have been described in the literature [7]. These procedures allowed the implant rehabilitation in situations that implant placement would be contraindicated in the past [8]. Several surgical techniques have been advocated for vertical bone augmentation of severely resorbed ridge, such as guided bone regeneration combined with bone graft [9], the interposition of bone block grafts (inlay technique) [10], sinus elevation [11, 12], and distraction osteogenesis [7]. The inferior alveolar nerve lateralization and transposition are the examples of uncommon procedures in the mandible [6]. In this scenario, the placement of short implants appears as an alternative treatment to avoid advanced surgical procedures and their corresponding morbidity [13-15].

Implants ≤10 mm with traditional machined surfaces showed inferior success rates compared with longer implants in the past [16-18]. Despite these early disappointing results of these implants, they continued to be used and improved. New technologies and knowledge have been resulting in improvements of implant surfaces, such as the modulation of osteoblasts adhesion and spreading induced by structural modifications of the titanium surface, and these changes have promoted an enhanced bone formation around implants [19-24]. The new concepts were also applied to the short implant surfaces, increasing their long-term clinical success [4]. Currently, it is already possible to find 4mm long implants, which have been evaluated in a multicenter single-cohort prospective study with promising results [25].

The use of short implants in oral rehabilitation is certainly a simpler, cheaper and faster treatment with less associated morbidity compared with longer implants placed in the augmented bone area [26]. However, it remains unclear the long-term survival rates of short implants models measuring less than seven millimeters in length. Few Randomized Controlled Trials (RCTs) comparing the effectiveness of prostheses supported by either short implants or longer implants placed in the augmented bone area for at least one year of follow-up were found in the literature [8, 26-31].

In order to establish the long-term success of short implants with 7 mm or less of length, the present systematic review compared the survival rate and the marginal bone loss between short implants placed in the atrophic posterior area and longer implants placed in the augmented bone area, with a one-year post-loading follow-up.

## 2 MATERIALS AND METHODS

### 2.1 Protocol and Registration

The present systematic review was registered in the International Prospective Register of Systematic Reviews ‘PROSPERO’ [32]. The protocol can be assessed at: http:// www.crd.york.ac.uk/ PROSPERO/ display_record.asp? ID=CRD42015015864, under the registration number: CRD42015015864.

This review was also conducted in accordance with the Preferred Reporting Items for Systematic Reviews and Meta-Analysis (PRISMA) [33].

### 2.2 Eligibility Criteria, Information Sources and Search Strategy

An advanced mode of electronic search was performed in the MEDLINE (PubMed), SCOPUS and Cochrane Library databases, up to May 2^nd^ 2017, to obtain studies related to short dental implants. In order to identify the studies to be included in the present review, the definition of the PICOS question (P=Patient; I=Intervention; C=Control; O=Outcome; S=Study design) was used to guide the following search strategy: P- patient who received dental implants; I- short implants (≤7 mm); C- longer implants (>7 mm); O- survival rate and peri-implant marginal bone loss; S- Randomized Clinical Trials (RCTs), retrospective and prospective studies.

The literature search strategy used in MEDLINE (PubMed) was [dental implants (MeSH Terms) or dental implant, dental implantation, endosseous dental implantation, endosseous implant, endosseous implantation, endosseous implants, oral dental implant, oral dental implants, or oral dental implantation] and [short* or short-length or short or short length OR length] and [success or survival or fail or failure] and [randomized controlled trials or retrospective or prospective]. The search terms applied for both Cochrane database and SCOPUS were dental implants, dental implant, dental implantation, endosseous dental implantation, endosseous implant, endosseous implantation, endosseous implants, oral dental implant, oral dental implants, oral dental implantation, short-length, short, short length, length, success, survival, fail, failure, randomized controlled trials, prospective and retrospective.

### 2.3 Study Selection (Inclusion and Exclusion Criteria)

Studies were selected by title and abstract for screening according to these inclusion criteria: studies with at least one year of follow-up; implants with 7 mm or less of length; studies with survival, success and failure rates; studies in adult humans.

Eligibility was based on full-text assessment using the following exclusion criteria: no information regarding the short implant sample; studies not related to short implants; non-RCT study; studies testing implant with more than 7 mm of length; the ones not mentioning bone loss in millimeters, and studies treating patients with fixed full dentures or overdentures.

### 2.4 Data Collection Process and Data Items

The literature review was independently conducted by three examiners (F.I., P.N. and V.H.M.). Inter-examiner reproducibility was 0.87 (Cohen´s Kappa) and a new calibration was performed to resolve any disagreement. Discrepancies and doubts were settled by data checking and discussion. When these discrepancies were not resolved by consensus, a fourth examiner (M.K.M) was consulted. In case of any missing data, the authors of the identified articles were contacted to provide any further details.

Data extracted from each of the included randomized controlled trials referred to the year of publication, the study design (RCT details), the methodology of the study (number of patients treated, implants placed and characteristics of each group of study), the outcome measures, results (failures and marginal bone level changes), conclusions, and others additional information.

### 2.5 Risk of Bias Assessment

A quality assessment of the studies included in the meta-analysis was performed following the recommendations for systematic reviews of interventions of the Cochrane collaboration [34]. The Cochrane Collaboration’s tool for assessing the risk of bias in randomized trials was used to identify studies with intrinsic flaws in the method and design. The risk of bias assessment focused on the following criteria: blinding of participants and personnel (performance bias), random sequence generation and allocation concealment (both accounting for selection bias), blinding of outcome assessment (detection bias), incomplete outcome data (attrition bias), selective reporting (reporting bias), or other possible causes of bias.

The risk of bias of each study was categorized according to the following criteria: low risk (plausible bias unlikely to seriously alter the results); unclear risk (plausible bias that raises some doubt about the results); high risk of bias (plausible bias that seriously weakens confidence in the results).

### 2.6 Summary Measures (Data Synthesis) and Statistical Analysis

Meta-analysis from the data extracted was performed using the Rev Man software, version 5.3 (The Nordic Cochrane Center, The Cochrane collaboration, Copenhagen, Denmark). The significance of treatment effects was tested using a fixed-effects model in the absence of a statistically significant heterogeneity. In turn, a random-effects model was used in case of high heterogeneity among the randomized controlled trials. Cochran Q test was performed (*p*<0.001/ CI 95%) to evaluate the heterogeneity among the studies and the presence of heterogeneity was analyzed using inconsistency test I^2^. The value of I^2^ ranged from 0 to 100, with larger values (≥75%) suggesting high heterogeneity. For continuous data elements such as marginal bone changes, the Mean Difference (MD) was calculated. For dichotomous data, such as the implant failure rate, a Risk Difference (RD) with the 95% CI was used to pool the results of each treatment group. The pooled effect was considered significant if *p*<0.05. A funnel plot was used to assess the presence of the publication bias. Sensitivity analysis was performed on primary outcome (survival rate).

## 3 RESULTS

### 3.1 Study Selection

**Fig. (1):**
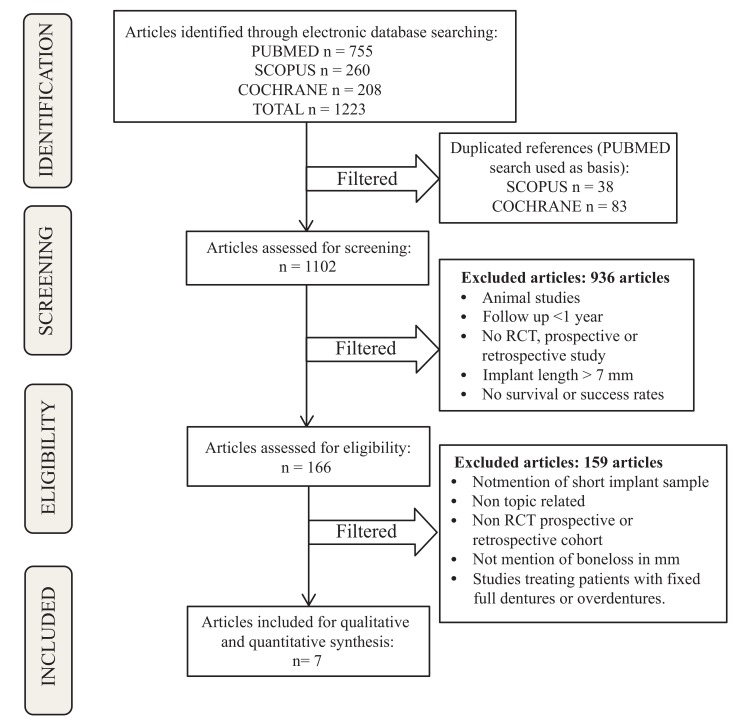
Flow diagram (PRISMA format) of the screening and selection process.

The systematic search displayed 755, 260 and 208 results from PubMed, Scopus and Cochrane Library databases, respectively. The preliminary exclusion was performed by duplicated references (n=38 from Scopus and n=83 from Cochrane database). One thousand one hundred and two studies were screened, analyzing the titles and abstracts of each one. A total of 936 articles were excluded in the screening phase. One hundred and sixty-six articles were assessed for eligibility. Studies that did not meet the aforementioned inclusion criteria were excluded in this phase (n=159). At the end of this process, seven RCTs were included in the review for qualitative and quantitative analyzes (Fig. **1**). Multiple studies have been published using data from the same population in different follow-up periods [26, 28, 35, 36]. Within this group of studies, only the one-year follow-up studies were included in the present review [26, 28].

### 3.2 Study Characteristics

A total of seven randomized controlled trials met the inclusion criteria (Table **1**) and were included in the meta-analyses. All studies had at least one year of follow-up after loading the implants with single crowns or fixed partial dentures (three elements maximum), in the posterior regions of mandibles and maxillae. Studies using full fixed dentures or overdentures to rehabilitate edentulous patients were excluded from this review as splinting anterior and posterior implants together could interfere with the survival rate of short implants in atrophic posterior regions.

### 3.3 Risk of Bias

**Fig. (2):**
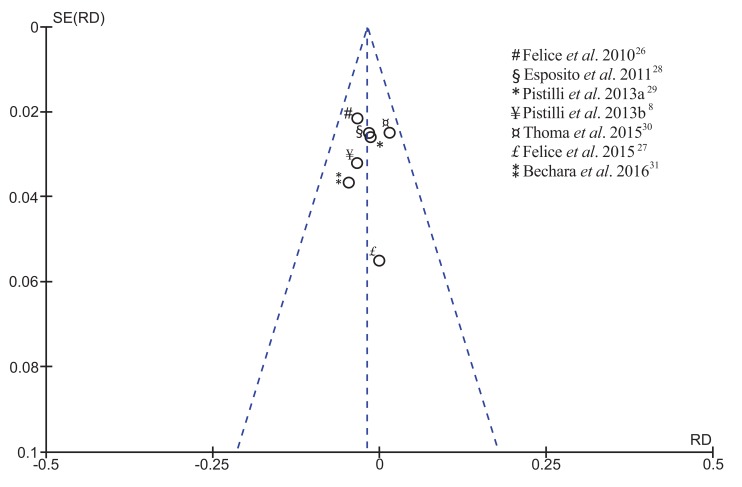
Funnel plot assessing the publication bias of studies included.

The results of publication bias showed the symmetric distribution for all RCTs qualitatively assessed, indicating low potential for the risk of bias, as illustrated in the funnel plot (Fig. **2**). The similarity of study design within the included articles may explain their homogeneous distribution.

As shown in Fig. (**3**), the performance bias of all included studies was unclear. Although the authors reported patients and operators blinding, it is impossible to blind the surgeon completely, especially in the split-mouth design studies. The ‘blinding of outcome assessment’ was considered a high risk of bias, as the radiographic images clearly expose the difference between the test and control groups, being unfeasible to blind the outcome assessors. All the studies were classified as low risk for all the other criteria of bias.

### 3.4 Meta-Analysis

The total of lost implants, short and longer ones, was used to calculate the survival rate for both test and control groups. The average of bone loss around the implants was shown as a mean and Standard Deviation (SD).

#### 3.4.1 Survival Rate (Risk Difference)

**Fig. (4):**
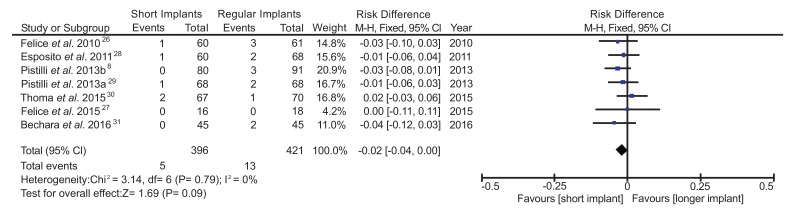
Forest plot for the event ‘implant survival rate’, comparing short implants (≤7 mm) placed in the atrophic area and longer implants placed in the augmented bone area of maxillae and mandibles.

Data of survival rate of short implants were considered dichotomous for the meta-analysis and it was assessed only for one-year of follow-up post-loading. Mantel-Haenszel method was used as the statistic method with fixed effect, using Risk Difference (RD) and 95% of Confidence Interval (CI). The RD between the short implant group and the control group was -0.02 (95% CI, -0.04 to 0.00), I^2^=0 and Chi^2^=3.14, indicating a lower risk of implant loss in the short implant group compared with the control group, although no statistical significance (*p*=0.09) was observed (Fig. **4**). The study of Felice **et al**. [27] showed the highest standard deviation, 95% CI -0.11 to 0.11). This might be a result of the limited number of patients as only 10 patients were allocated in each group of study in his study. All the other RCTs included in this review were conducted with more than 30 patients (Table **1**). Opposing to all other studies, the RCT of Thoma **et al**. [30] was the only one presenting a higher survival rate for long implants placed in augmented bone area than for short implants. As a result, no statistical difference between the test and control groups could be identified in our meta-analysis, although the sensitivity analysis demonstrated that Thoma’s study had no influence on the RD.

### 3.5 Marginal Bone Loss (Mean Difference)

The bone loss outcome used continuous data for the statistical analysis and the method of analysis was the inverse variance with random effect. Mean bone loss was analyzed according to the original data. The mean difference was -0,13 (95% CI -0.22 to -0.05), I^2^ = 46% and Chi^2^ = 7.39, indicating that the marginal bone changes were lower in the short implant group compared to the control group. The studies showed more heterogeneity for this outcome in comparison with the survival rate. In Fig. (**5**), a favorable result for the test group can be noticed with a significant statistical difference between the groups (*p*=0.002). Two studies [30, 31] were excluded of bone loss analysis as they did not furnish enough information regarding the bone level changes in their samples to be eligible for meta-analysis.

## 4 DISCUSSION

The present systematic review is the first study to make a meta-analysis of the survival rate and marginal bone loss around short implants (≤7 mm) compared with longer implants placed in the bone grafted area. The absence of consensus regarding the definition of short implants makes difficult the classification of short implants according to their length [4, 19, 22, 29]. Although implants measuring 7-10 mm of length have already been considered short implants by some authors [14, 37, 38], this systematic review considered implants ≤7 mm long as short implants as previous studies reported higher failure rate of 7 mm implants [16] and information about the success of these short implants is still scarce.

All included studies presented both performance and detection biases because the evident physical difference between regular and short implants makes impossible to blind the operators and the outcome assessors during the surgery, and the clinical and radiographic examination [39]. However, these biases may be considered intrinsic to the methodology in this type of study and do not weaken the confidence in the results.

A total of seven studies [8, 26-31] presented similar methodologies, especially regarding the inclusion criteria and allocation of patients (Table **1**). The same pattern of pre and postoperative cares, surgical technique and bone augmentation procedures observed in all studies corroborates with the consistency of the analysis of the results. A total of 396 short implants placed and 421 longer implants were included in the present review. Out of the total of implants placed, 5 short implants and 13 longer implants were lost with meta-analysis data showing RD= -0.02 (95% CI, -0.04 to 0.00). This result translates into a reduction of the risk of implant failure using short implants by 49% relative to longer implants within the first years of loading.

Previous studies reported higher failure rates for short implants in comparison with long implants [16-18, 40-42]. On the other hand, in the present systematic review no significant difference (*p*>0.05) on the survival rate between short and regular implants placed in augmented bone area (98.7% and 96.9%, respectively) was identified, despite the slight tendency for a better survival rate in favor of short implants after one year of implant loading. These results are in accordance with previous studies of Friberg **et al*.* [43] and Lekholm **et al*.* [41], which reported 92,3% e 93,5% of survival rate, respectively, after 10 years of short implants placement.

**Fig. (5):**
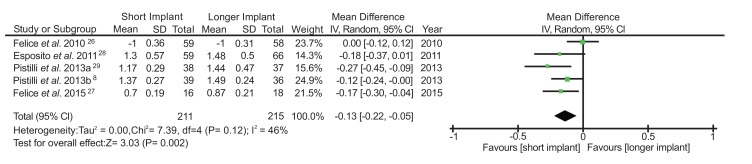
Forest plot for the event ‘marginal bone loss’. Data collected after one-year post-loading.

Bone loss around short implants is extremely sensitive to the implant treatment success as one millimeter of marginal bone loss in an implant shorter than 8 mm represents 12.5% of bone support loss [44]. A significant difference of bone loss (*p*=0.02) between short and longer implants was observed in our analysis (Fig. **5**). We found that implants ≤7 mm long present less bone loss than longer implants placed in augmented bone area after one-year of prostheses placement under occlusal forces. In a previous systematic review [21], the average bone loss around short implants was 0.83 mm after 4 years of follow-up. On the other hand, another study conducted with 126 patients [45] reported that 72% of short implants presented no bone loss after 6 years of follow-up, despite an average of 0.2 mm of additional peri-implant marginal bone loss is expected between the first and third year after the implant placement.

As bone loss around implants with limited length may compromise the success of the oral rehabilitation treatments, and consequently the patient’s oral health, there is a demand for long-term clinical evaluation (at least 5 to 10 years) of short implants ≤7 mm long to demonstrate that they are a better treatment option than augmentation procedures followed by long implant placement.

Many factors influence the survival rates and implant success. The studies included in our meta-analysis accepted smokers in their assessments, although smoking has been reported as an important factor of risk associated with patient for implant treatment success [46, 47]. Related to the implant structure, the implant surface treatment, such as the incorporation of calcium ions, is another factor that significantly influences the implant osseointegration and its bone loss [19, 22, 23,48]. Implant geometry and surface topography also play an important role in the success of implants shorter than 7 mm [19], possibly enabling the achievement of survival rates equivalent to longer implants [49].

The position of the short implants in the arch is considered a major factor for the implant longevity. Mezzomo **et al*.* [21], using meta-analysis, reported that short implants placed in the mandible had a lower incidence of implant failures/complication and marginal bone loss than maxillary implants, but this result is not a consensus [50, 51]. The incidence of failures and complications associated with short implants supporting single crowns or fixed partial dentures in posterior regions of maxillaries cannot be influenced by the bone quality and implant length per se [21], so the present systematic review included primary studies assessing implants placed in both maxillae [30] and mandible [26]. Hence, our results cannot be extrapolated to anterior implants as only posterior implants were evaluated.

A well-conducted prosthodontic rehabilitation is essential for short implant longevity. Occlusal overload, inadequate crown/implant ratio, non-splinting crowns, presence of cantilever are examples of factors associated with implant-supported prosthesis that can lead to marginal bone loss and should be avoided [21, 38, 40, 52, 53].

During oral rehabilitation of atrophic areas, the avoidance of bone augmentation procedures reduces discomfort, treatment time and costs for the patient ^26^. All these factors make the placement of short implants an attractive choice of treatment for both patient and clinician if the success rates are not substantially decreased. According to our meta-analysis, short implants presented similar success rates (survival rate and bone loss) to longer implants in grafted bone area after one-year of loading. Despite these favorable results of short implants for the rehabilitation of atrophic jaws, precautions should be exerted when interpreting the results of this review. The heterogeneity of methodology (*e.g.* different implant brands) between the studies and the small number of clinical trials with long-term follow-up available in the literature limit the conclusion that short implant placement is a better choice of treatment than long implants placed in the augmented bone area. Moreover, further studies are necessary to evaluate the impact of prosthodontic rehabilitation on the survival rate of short implant in the long-term.

## CONCLUSION

Short implants (≤7 mm) had a survival rate similar to longer implants placed in the augmented bone area after one-year post-loading, however, the marginal bone loss at short implants sites was lower than the longer implants sites. Placement of short implants is a predictable alternative for the rehabilitation of atrophic posterior regions, avoiding all the disadvantages intrinsic to bone augmentation procedures.

## Figures and Tables

**Fig. (3):**
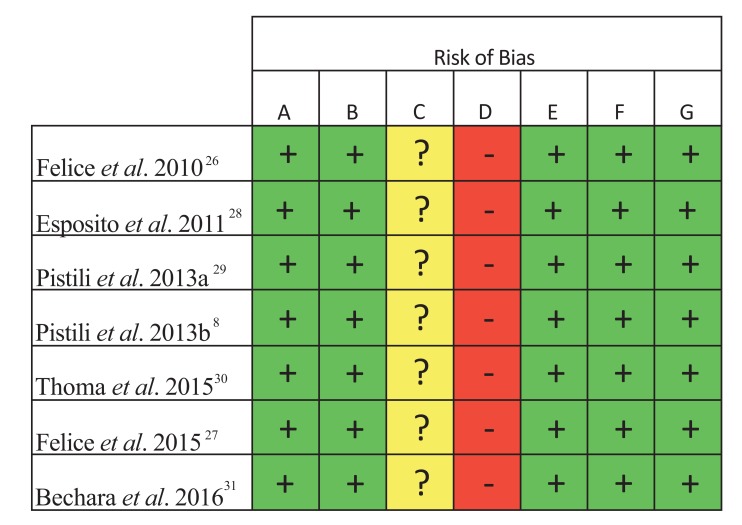
Assessment of the risk of bias of studies included. Low risk of bias (+), unclear risk of bias (?) and high risk of bias (−).

**Table 1:** Main characteristics of the studies selected.

**Study**	**Study Design**	**Population**	**Implants Brand**	**Implants Number (size length x width)**	**Augmentation Procedure**	**Results**	**Conclusions**
**Felice **et al*.* 2010 ^26^**	RCT	N = 60 patients F/M gender ratio: 38/22 Age range: 40-83 years	NanoTite - Biomet 3i (Palm Beach, USA))	60 short (7 x 4 mm) 61 longer (10, 11.5, 13, 15 x 4 mm)	Vertical augmentation of mandibles with inorganic bovine bone blocks.	IF: 3 Longer and 1 Short MBL: Short 1.79 + 0.54 mm Longer 1.65 + 0.42 mm	Interpositional bovine block grafting and placement of short implants achieved good and similar results. Short implants might be a preferable choice when the bone height is limited as the treatment is faster, cheaper and with less morbidity.
**Esposito **et al*.* 2011 ^28^**	RCT (split mouth design	N = 30 patients F/M gender ratio: 17/13 Age range: 37-70 years	MegaGen Implant Co. (Gyongbuck, South Korea)	60 short (5 x 6 mm) 68 longer (10 x 6 mm)	Interpositional bone blocks in mandibles or particulated bone in augmented maxillary sinuses.	IF: 2 Longer and 1 Short MBL: Short: 1.30 + 0..57mm Longer: 1.48 + 0.50 mm	All techniques provided good and similar results up to 1 year after loading, however, 5 mm short implants might be a preferable choice to augmentation procedures
**Pistilli **et al*.* 2013a ^29^**	RCT (parallel group design	N = 80 patients F/M gender ratio: 55/25 Age range: 57-75 years	MegaGen Implant Co. (Gyeongbuk, South Korea)	68 short (5 x 5 mm) 68 longer (11.5, 13, 15 x 5 mm)	Equine bone blocks in mandibles or particulated porcine bone in augmented maxillary sinuses.	IF: 2 Longer and 1 Short MBL: Mandible – Short: 1.18+ 0.29mm Longer: 1.36 + 0.28mm Maxilla – Short: 1.16 ± 0.30 mm Longer: 1.53 ±0.59 mm	One year post loading, 5 x 5mm implants achieved similar (in the maxilla) if not better (in the mandible) results than longer implants placed in augmented bone
**Pistilli **et al*.* 2013b ^8^**	RCT (split mouth design)	N = 40 patients F/M gender ratio: 19/21 Age range: 55-85 years	Southern Implants (Irene, South Africa)	80 short (6 x 4 mm) 91 longer (≥10 x4 mm)	Equine bone blocks in mandibles or particulated porcine bone in augmented maxillary sinuses.	IF: 3 Longer MBL: Mandible – Short: 1.33 + 0,22 mm Longer: 1.44+ 0,21mm Maxilla – Short: 1.41 + 0,31 mm Longer: 1.53 + 0,29 mm	Short implants may be as effective, if not more effective, than longer implants placed in augmented posterior jaws
**Thoma **et al*.* 2015 ^30^**	RCT (parallel group design	N = 101 patients F/M gender ratio: 52/49 Age range: 20-75 years	Astra Tech (Dentsply Implants, (Mölndal, Sweden)	67 short (6 x 4 mm) 70 longer (11-15 x 4 mm)	Sinus lift procedure using particulated bovine bone material.	IF: 1 Longer and 2 Short	Both treatment modalities are safe and successful rendering a high implant survival rate.
**Felice **et al*.* 2015 ^27^**	RCT	N = 20 patients F/M gender ratio: 12/8 Age range: 43-70 years	Zimmer Biomet (Florida, USA)	16 short (5-6 x 5 mm) 18 longer (10 x 6 mm)	Sinus lift procedure using granular inorganic bovine bone substitute.	IF: none MBL (one year after loading): Short: 0.70 ± 0.19 mm Longer: 0.87 ± 0.21 mm	Both techniques achieved excellent and similar results.
**Bechara **et al*.* 2016 ^31^**	RCT	N=53 patients F/M gender ratio: 34/19 Age range: 21-76 years	MegaGen Implant Co (Gyeongbuk, South Korea)	45 short (6 x 4-8 mm) 45 Longer (10-, 11.5-, 13-, or 15-mm x 4-8 mm)	Sinus lift procedure using a collagenated porcine particulate bone graft.	IF: 2 Longer MBL (mean): 1 year – Short: 0.14 mm Longer: 0.21 mm 3 years – Short: 0.20 mm Longer: 0.27 mm	Both treatment modalities showed similar results. Short implants might be preferable, because the treatment is faster and less expensive.

RCT =Randomized clinical trial ; F/M= female/male; Implant failure = IF; Marginal bone loss = MB

## ABBREVIATIONS

RCT: Randomized Controlled Trial
RD: Risk Difference
CI: Confidence Interval
Mm: Milimiter
MD: Mean Differences
